# Multi-Component Systems Remaining Useful Life Prediction Integrating Physical Prior and Variational Spatiotemporal Networks

**DOI:** 10.3390/s26217031

**Published:** 2026-11-06

**Authors:** Changjun Li, Jinjun Cheng, Haizhen Zhu, Bincheng Wen, Ziyi Yu

**Affiliations:** Air Force Engineering University, Xi’an 710038, China; lylcj615116@163.com (C.L.);

**Keywords:** physical prior, variational spatiotemporal network, aero-engine, remaining useful life prediction

## Abstract

Modernized equipment features a multi-component structure, which is characterized by complex degradation behaviors, noisy monitoring measurements, and considerable uncertainty in life prediction, making accurate remaining useful life (RUL) forecasting a challenging task. To address this issue, this paper takes an aero-engine as a typical research subject and proposes a multi-component systems RUL prediction method that integrates physical priors and a variational spatiotemporal graph convolutional network. Firstly, graph-structured data are constructed according to the physical topological relationships among multiple engine components, and an initial adjacency matrix is generated to endow the model with prior physical knowledge of sensor dependencies. Secondly, a spatiotemporal feature extraction module is established, which combines graph convolution, dual attention mechanisms, and temporal convolution to adaptively select informative sensor channels and critical spatiotemporal features. Finally, a variational information bottleneck mechanism is introduced. By means of mask-based noise reduction and dynamically learned probabilistic graph structures, information relevant to life-related characteristics is screened, and the processed features are fed into a multi-layer perceptron to implement RUL regression prediction. Validations on the public C-MAPSS aero-engine dataset demonstrate that the proposed method adequately combines the merits of physical priors and data-driven paradigms. It effectively mitigates noise interference and reduces prediction uncertainty, and can provide technical support for health management and life prediction of multi-component aero-engine systems.

## 1. Introduction

With the advancement of intelligent manufacturing technologies, modernized equipment is evolving toward high complexity and high intelligence in structure and functionality, which poses greater challenges to the safety and reliability of system-level devices [1,2]. Prognostics and Health Management (PHM) technology enables health-condition monitoring and predictive maintenance for equipment systems, providing a technical approach for low-cost maintenance of complex system devices [3]. As a core technology of PHM, RUL prediction aims to exploit historical operational data collected by multi-sensor systems and establish the mapping relationship between sensor measurements and equipment lifetime to achieve RUL estimation of system devices [4]. An aero-engine is a typical highly complex, long-lifespan multi-component coupled system. Its components form tight physical interactions through mechanical connections, thermal conduction, and airflow coupling. During the service period, aero-engines generally operate under extreme working conditions such as high speed, high temperature, and high pressure, exhibiting typical spatiotemporal coupled degradation characteristics of multiple components [5]. Therefore, accurately exploring the coupled degradation mechanism of multi-components and realizing high-precision RUL prediction for aero-engines constitute a critical component of the prognostics and health management system for aviation equipment.

At present, aero-engines are commonly equipped with multiple sensors to collect various physical parameters, including temperature, pressure, rotational speed, and fuel flow for real-time operational condition monitoring. Nevertheless, the massive multi-source sensor data are characterized by high dimensionality, strong coupling, strong nonlinearity, and time-varying properties. Effectively extracting degradation-relevant information from massive sensor data and constructing accurate RUL prediction models are essential for precise RUL estimation of aero-engines. Conventional RUL prediction approaches can be mainly divided into two categories: model-driven methods [6,7] and pure data-driven methods [8,9]. Model-driven methods construct degradation equations based on physical mechanisms such as thermodynamics, fluid mechanics, and fatigue damage, and perform quantitative characterization using physical structural parameters and operational condition parameters. However, such methods suffer from high modeling difficulty and poor generalization performance, making them difficult for degradation modelling of complex multi-component coupled equipment [10]. Representative pure data-driven approaches include temporal models such as deep neural networks, convolutional neural networks, and long short-term memory neural networks [11,12,13,14], which extract degradation features from sensor data via nonlinear mapping to implement aero-engine RUL prediction.

Such methods neglect the inherent physical topological connections among equipment components as well as the spatial positional dependencies of sensors, and therefore cannot accurately characterize the composite degradation features induced by multi-component coupling. In recent years, graph neural networks (GNNs) have incorporated physical association information between sensors or components into neural network prediction models through non-Euclidean structure modeling, providing a new perspective for breaking through the above limitations. To date, researchers have applied various GNNs and their improved variants to aero-engine RUL prediction. Meng et al. [15] proposed a fault diagnosis method that integrates modal analysis with a graph attention network. By extracting phase information at different scales and orientations from sensor data, a novel graph-structured input was constructed, and physical information was embedded into the graph structure. Compared with conventional GNNs, this method improved the accuracy of damage identification from vibration sensors. To capture the latent dynamic relationships among sensor data, Wang et al. [16] proposed a comprehensive dynamic graph structure network that combines the visibility graph algorithm with a graph convolutional network to extract global temporal features. By integrating the structural characteristics of the engine with dynamic dependencies, a comprehensive graph structure was generated, which improved the RUL prediction accuracy of conventional GNNs. However, this method remains insufficient in terms of data uncertainty and information fusion theory. Yu et al. [17] proposed a novel fault diagnosis framework based on GNNs and a dynamic graph embedding mechanism. By designing a dynamic graph edge dropout mechanism, GNNs were applied to extract the sensitivity of fault features to operating conditions, achieving-domain adaptive fault diagnosis of bearings under variable working conditions. Considering the influence of equipment component structural data and prior knowledge, Zhang et al. [18] proposed a temporal knowledge graph information network that combines prior knowledge with structural data to accurately characterize spatiotemporal features. Wu et al. [19] designed a temporal multi-resolution hypergraph attention network to address the insufficient interdependence among the monitoring signal samples of rolling bearings. To overcome the limitations of conventional graph structures in terms of temporal and frequency domain prior knowledge, Cui et al. [20] proposed an RUL prediction method that fuses a sparse graph structure with a convolutional network. By designing a node-level graph structure and converting time series into node features, the capability of the graph structure to represent mechanical degradation information was significantly enhanced. Gao et al. [21] proposed a multi-sensor universal spatiotemporal convolutional network that captures spatiotemporal features from structured data through a dual graph attention module, enabling first-time prediction time detection. To address the issue that existing RUL prediction methods fail to consider the inherent heterogeneous association properties of multi-sensor data, Xiao et al. [22] proposed a heterogeneous graph convolutional network with cross-temporal information propagation. By constructing a cross-spatio-temporal heterogeneous graph, spatio-temporal dependency information among multi-sensor variables was extracted, enhancing the spatio-temporal representation capability of RUL prediction. Although GNNs and their improved variants have demonstrated unique advantages in spatial modeling, existing methods rely heavily on data-driven statistical correlations in graph structure construction and lack explicit representation of the physical connections of aero-engines. To remedy this deficiency, physics-informed GNNs have gradually become a research hotspot. Cui et al. [23] encoded thermodynamic principles into the adjacency matrix construction process, realizing physics-aware graph structure initialization. Zhang and Amin et al. [24,25] incorporated physical information into aero-engine RUL prediction by designing a static-dynamic hybrid graph neural network model. Zhou et al. [26] employed a thermal cycling physical model as prior knowledge to construct a spatial-domain data association graph. However, current physical information fusion mostly remains at the soft constraint level rather than treating physical topology as a prior for graph structure construction, which limits the generalization performance of the models. Besides graph neural networks, state-of-the-art time series modeling approaches have also advanced the development of RUL trajectory modeling. The bidirectional Mamba model built upon the state space model possesses powerful capability for long sequence modeling [27], and has been adopted for aero-engine remaining useful life prediction to effectively capture complete degradation trajectories. Nevertheless, most existing Mamba RUL studies only model time series signals and rarely incorporate physical topological priors of components into the network. Furthermore, causal discovery techniques provide a new perspective to address spurious edges in graph models. Such techniques aim to identify genuine causal degradation relationships between sensors and components, so as to replace graph construction based on simple statistical correlation. However, for high-dimensional multi-sensor aero-engine scenarios, causal discovery algorithms suffer from high computational complexity, which brings considerable challenges for online deployment. In terms of complex mechanical degradation assessment, multi-mode degradation evaluation and combined vibroacoustic monitoring for aerospace harmonic drive systems have attracted increasing research attention [28]. Vibroacoustic signals can reflect multi-mode coupled damage, yet how to deeply fuse such monitoring data with physical topology-aware graph models still requires further in-depth investigation. In terms of uncertainty quantification and information filtering, variational methods and information bottleneck theory have provided new theoretical tools for RUL prediction. Li et al. [29] designed a variational information bottleneck graph filter that models node features as Gaussian distributions, quantifies uncertainty using variance, and adaptively suppresses high-noise nodes through an information bottleneck gate. Liu et al. [30] proposed a graph learning framework based on a variational graph-structure information bottleneck. By inferring the probability distribution of the graph structure, they were able to retain task-related dependencies and suppress redundant connections, achieving a prediction error reduction of 2.9~14.8% on multi-sensor equipment datasets. Zheng et al. [31] compressed redundant information in graph representations through a hierarchical subgraph neural network and an information bottleneck module. In addition, a mechanical multi-sensor RUL prediction method based on the variational graph structure information bottleneck has also verified the effectiveness of this paradigm in multi-sensor scenarios [30].

In summary, existing RUL prediction methods for multi-component systems still face the following key challenges: (1) Lack of physical priors in graph topology construction. Most methods rely on data-driven correlation-based graph construction or learnable implicit graph structures, without incorporating physical prior information such as real mechanical connections or thermal coupling among components. This tends to generate a large number of spurious association edges and results in poor model interpretability. (2) Graph topology structure solidification. The constructed graph structures mostly adopt static fixed adjacency matrices, which cannot adapt to the characteristic that component coupling strengths dynamically evolve with the degradation process during real service, making it difficult to characterize dynamic degradation associations. (3) Insufficient feature selection capability. Multi-layer graph convolution causes node features to tend toward homogenization, thereby erasing the degradation differences among different components. Sensor data contain substantial noise and operating-condition redundant features that are weakly correlated with RUL, while conventional GNNs lack an effective information filtering mechanism. (4) Lack of uncertainty modeling capability. Existing physical information fusion is mostly implemented in the form of soft constraints, and its integration with uncertainty quantification and feature compression methods such as variational inference and information bottleneck remains insufficient. To address the above challenges, this paper takes an aero-engine as the research object and proposes a multi-component system RUL prediction model based on the fusion of physical priors and variational spatiotemporal graph convolutional networks. By constructing graph-structured data with physical topology priors, the physical positional dependency information among sensors is extracted. A dual attention mechanism is employed to enhance the spatiotemporal feature representation capability of the model, and variational graph information bottleneck theory is introduced to optimize the feature filtering mechanism, thereby achieving accurate RUL prediction. The main contributions of this paper are as follows: (1) A physical prior-driven initial graph structure is constructed. Distinct from pure data statistics-based graph construction, the adjacency matrix is generated based on the real component coupling topology of an aero-engine, endowing the graph structure with explicit physical and engineering significance. This effectively eliminates spurious association edges and improves model interpretability as well as adaptability under small-sample operating conditions. (2) A dual attention spatiotemporal joint feature extraction strategy is proposed. By fusing graph convolution, a dual-attention mechanism, and dilated causal convolution, spatial physical coupling interaction, adaptive enhancement of key features, and mining of long-term temporal degradation patterns are accomplished simultaneously, addressing the issues of insufficient spatiotemporal feature extraction and weak noise anti-interference capability in conventional models. (3) A variational graph structure information bottleneck optimization module is designed. Through mask-based noise reduction, adaptive probabilistic graph updating, variational probabilistic modeling, and attention-based feature completion, redundant information compression, maximal retention of effective degradation information, and uncertainty quantification are realized, breaking through the technical bottlenecks of conventional deterministic graph models, namely rigid topology, susceptibility to overfitting, and loss of fine-grained features.

The remainder of this paper is organized as follows. Section 2 elaborates on the proposed model and method. Section 3 introduces the experimental dataset used for model validation. Section 4 analyzes the model prediction results, and Section 5 concludes this paper.

## 2. Model Method

**Figure 1:**
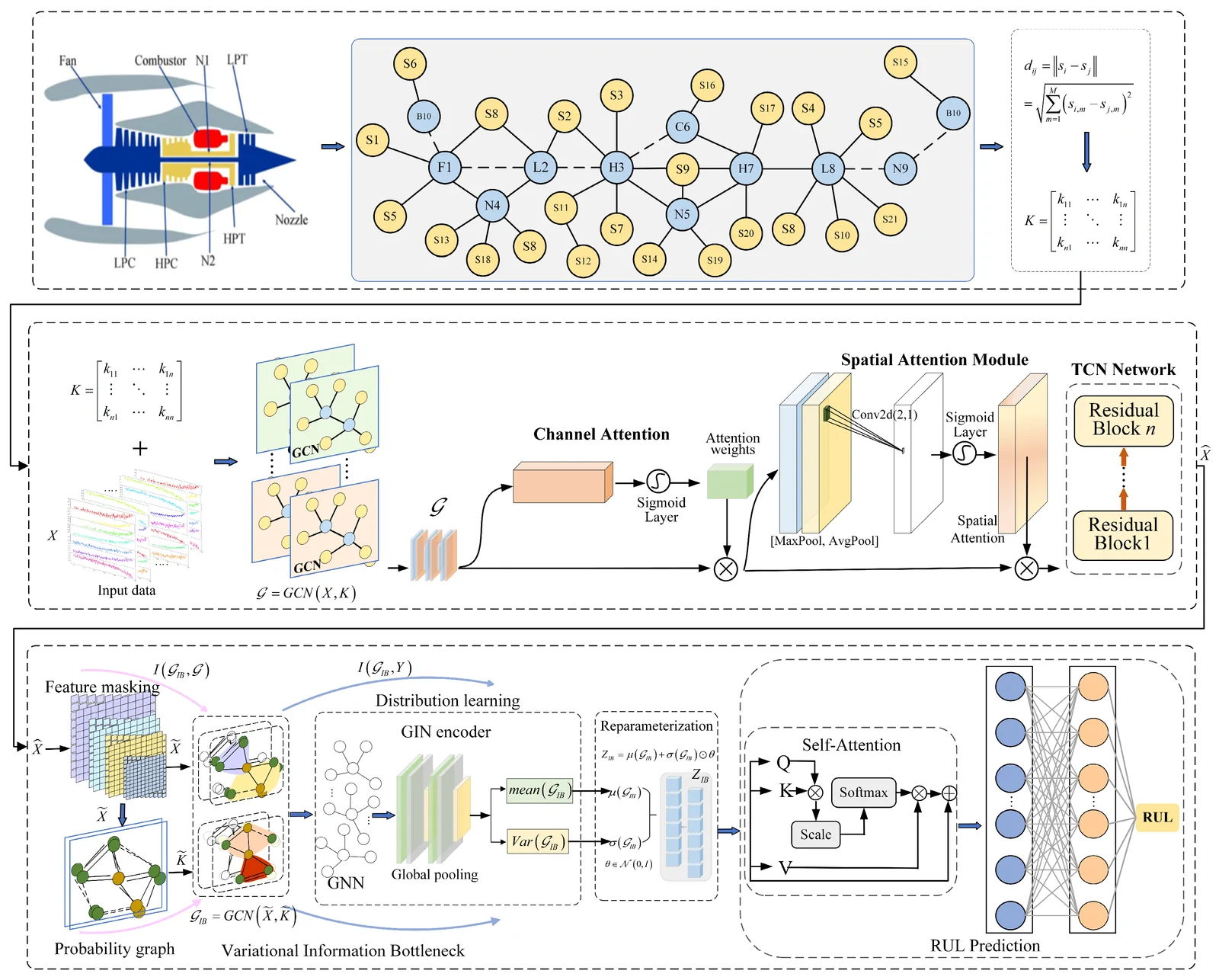
Research Approach.

Taking an aero-engine as an example, this paper conducts research on remaining useful life prediction for multi-component systems based on the fusion of physical priors and variational spatiotemporal graph convolutional networks. Figure 1 illustrates the overall research framework of this paper. The proposed prediction model mainly consists of three modules. The first one is the physical topology mapping and graph structure construction. Based on the multi-component physical connection structure of the aero-engine, multi-sensor data in non-Euclidean space are transformed into graph-structured data, and an initial adjacency matrix *K* is obtained. This provides topological relationship priors for the subsequent graph neural network, enabling the model to perceive the physical positional dependencies among different sensors. The second is the Spatiotemporal feature extraction. The obtained initial adjacency matrix *K* is subjected to graph convolutional network computation on the graph, such that the information of each sensor interacts with that of its physically adjacent or correlated sensors, generating a feature map 𝒢 that incorporates spatial information. Subsequently, channel attention is employed to perform global pooling on the feature map output by the GCN. Channel weights are generated through fully connected layers and activation functions, adaptively learning the sensor channels that are more important for the current degradation state while suppressing the interference of noisy channels. Furthermore, spatial attention is applied to compute the weights of spatial positions in the graph features, highlighting the spatial locations or temporal segments in the feature map that contribute most to RUL prediction, thereby further enhancing the spatial representation capability of the features. Finally, the spatial graph features enhanced by the dual attention mechanism are processed by temporal convolution. Causal convolution and dilated convolution are utilized to extract long-term temporal dependency features from sensor data and capture the temporal degradation patterns of equipment. Consequently, joint extraction of spatial graph dependencies and temporal degradation dependencies is achieved, completing the preliminary mapping from raw signals to high-dimensional spatiotemporal features. The third is the Variational graph structure information bottleneck and prediction. First, the initial graph features $\hat{X}$ processed by the spatiotemporal feature extraction module are subjected to mask processing to filter out node feature granularity information. Through the masking mechanism, irrelevant noise is removed, and only the feature subset strongly correlated with RUL is retained. Second, a probabilistic adjacency matrix $\tilde{K}$ is relearned based on the masked features to construct a probabilistic graph and generate a new graph structure 𝒢*_IB_*. A graph isomorphism network GIN encoder is then employed to extract graph features, encoding the entire graph structure information into Gaussian distribution parameters. The discrete graph is mapped into a continuous latent variable space, outputting two sets of parameters, namely the mean *μ*(𝒢*_IB_*) and the variance *σ*(𝒢*_IB_*). The latent variable *Z_IB_* is obtained by sampling via the reparameterization trick. By introducing a variational autoencoder, deterministic features are mapped into probability distributions, effectively capturing the uncertainty in the data, preventing model overfitting, addressing the issue that gradients cannot be backpropagated through random graph sampling, and enabling end-to-end differentiable training of the entire probabilistic graph model. Finally, the extracted key latent variables *Z_IB_* are fed into a self-attention mechanism to reinforce the long-range dependency degradation features within the latent variables, compensating for the fine-grained information loss caused by GIN global pooling. The attention-fused features are then input into an MLP to realize the regression mapping from deep representations to RUL scalars. The specific implementation method is described as follows.

### 2.1. Physical Topology Mapping and Graph Structure Construction

**Figure 2:**
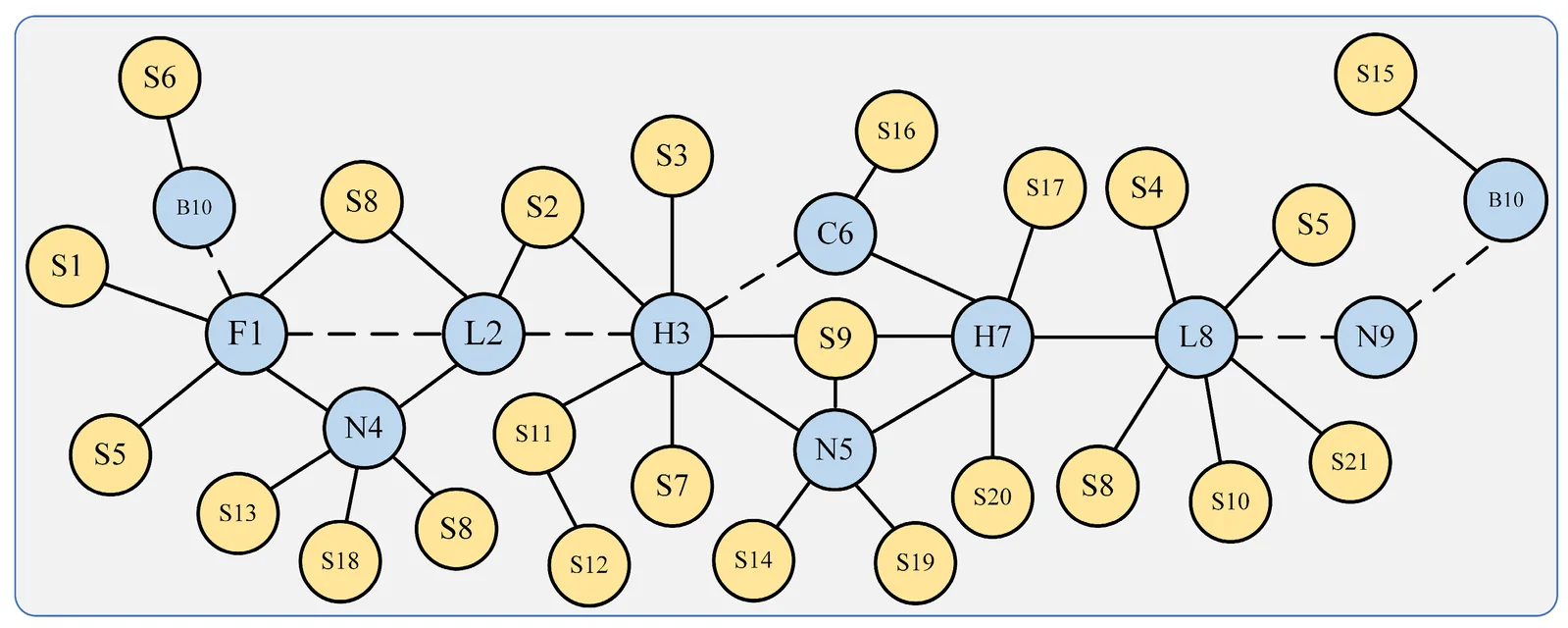
The schematic diagram of physical component connection and sensor layout for the aero-engine.

An aero-engine is a typical multi-component connected system, whose major components consist of the fan, low/high-pressure compressor, combustion chamber, low/high pressure turbine, inlet duct, and nozzle. The wear condition of each component directly affects the overall health status of the aero-engine. The interactions among different aero-engine components fall into three categories: direct connection, airflow-driven connection, and no connection. A direct connection refers to physical contact between two components, where their performance states are mutually correlated. An airflow-driven connection indicates that two components are coupled via gas flow, such as the fan and low-pressure compressor, and the low-pressure compressor and high-pressure compressor. Although these components are not physically attached to one another, intake airflow closely couples their performance behaviors. No connection means that two components exert no mutual influence. Figure 2 presents the schematic diagram of physical component connections and sensor layout for the aero-engine. The blue blocks denote the physical components of the aero-engine, and the yellow blocks represent the sensors deployed for corresponding physical components. F1 denotes the fan, L2 the low-pressure compressor, H3 the high-pressure compressor, N4 the fan spool speed, N5 the core spool speed, C6 the combustion chamber, H7 the high-pressure turbine, L8 the low-pressure turbine, N9 the nozzle, and B10 the bypass duct. Solid lines in the figure stand for direct physical connections between components, while dashed lines represent airflow-driven connections.

Let *HI* denote the health index of the aero-engine during operation. According to the connection characteristics of its major components, *HI* = {*F*1,*L*2,*H*3,*N*4,*N*5,*C*6,*H*7,*L*8,*N*9,*B*10}, where {*F*1,⋯,*B*10} represents the functional components of the aero-engine. Nevertheless, in practical operation, each functional component is mainly monitored via parameters collected by sensors, {*S*1,⋯,*S*21}. Since some functional components are mutually correlated, information redundancy exists in the corresponding sensor measurements. For instance, S8 denotes the fan rotational speed of the engine, which reflects the performance status of the fan and meanwhile exerts influences on the low-pressure compressor. Combined with the structural components and sensor layout of the aero-engine, the mathematical expressions describing the correlations among components as well as between components and sensors are formulated as:

(1) $$\begin{array}{l} F1=\{L2,N4,B10,S1,S5,S8\}\\ L2=\{F1,N4,H3,S2,S8\}\\ H3=\{L2,N5,C6,S2,S3,S7,S9,S11,S12\}\\ N4=\{F1,L2,S8,S13,S18\}\\ N5=\{H3,H7,S9,S14,S19\}\\ C6=\{H3,H7,S16\}\\ H7=\{C6,N5,L8,S9,S17,S20\}\\ L8=\{H7,N9,S4,S5,S8,S10,S21\}\\ N9=\{L8,B10\}\\ B10=\{F1,N9,S6,S15\} \end{array}$$

Based on the correlation relationships among components and between components and sensors, an initial graph structure grounded on physical priors is constructed as: 𝒢_0_ = (*V*,*K*,*X*), where *V* is the node set, *V* = {*S*1,⋯,*S*21}, *K* is the edge set representing the topological connections among sensors. *X* is the sensor input data, *X* = {*x*_1_,*x*_2_,⋯,*x_T_*}, *X* ∈ *ℝ^T^*^×^*^N^*, where *T* is the time step size, and N is the number of sensor nodes (Input feature dimension). Let *s_i_* and *s_j_* denote the signal feature vectors of the *i* and *j* sensors, respectively. The Euclidean distance between node *i* and node *j* can be formulated as follows:

(2) $$\begin{array}{l} d_{\mathrm{ij}}=\left\|s_{i}-s_{j}\right\|\\ =\sqrt{\sum_{n=1}^{N}{\left(s_{i,n}-s_{j,n}\right)}^{2}} \end{array}$$

Assuming that the distance values of the solid lines in the physical topological connection structure between components and components, and between components and sensors are 1, the distance values of the dashed lines are 0.5, and the distance values of no connection are 0, the initial connection matrix between each component can be obtained, *A_s_* that is:

(3) $$A_{s}=\begin{array}{l} F1\\ L2\\ H3\\ N4\\ N5\\ C6\\ H7\\ L8\\ N9\\ B10 \end{array}\left[\begin{array}{llllllllll} 1 & 0.5 & 0 & 1 & 0 & 0 & 0 & 0 & 0 & 0.5\\ 0.5 & 1 & 0.5 & 1 & 0 & 0 & 0 & 0 & 0 & 0\\ 0 & 0.5 & 1 & 0 & 0 & 0 & 0 & 0 & 0 & 0\\ 1 & 1 & 0 & 1 & 0 & 0 & 0 & 0 & 0 & 0\\ 0 & 0 & 1 & 0 & 1 & 0 & 1 & 0 & 0 & 0\\ 0 & 0 & 0.5 & 0 & 0 & 1 & 1 & 0 & 0 & 0\\ 0 & 0 & 0 & 0 & 1 & 0 & 1 & 1 & 0 & 0\\ 0 & 0 & 0 & 0 & 0 & 0 & 1 & 1 & 0.5 & 0\\ 0 & 0 & 0 & 0 & 0 & 0 & 0 & 0.5 & 1 & 0.5\\ 0.5 & 0 & 0 & 0 & 0 & 0 & 0 & 0 & 0.5 & 1 \end{array}\right]$$

These weight values are determined based on the physical coupling mechanism of aero-engine components, where each weight characterizes the strength of degradation coupling between components. For components with direct physical connections, vibration and thermal damage are transmitted directly, yielding the strongest coupling effect; hence a weight value of 1 is assigned. For airflow-coupled components without solid-state contact, indirect state correlation is realized via working medium flow. Their coupling strength is weaker than that of direct physical connections, so a compromise value of 0.5 is adopted. Components with no physical interaction exert no mutual influence on each other’s degradation, and are therefore assigned a weight of 0. It should be noted that these values are only utilized for the initialization of the adjacency matrix. The subsequent model dynamically learns a probabilistic adjacency matrix through the variational graph structured information bottleneck module. The initial connection weights are adaptively updated during training, and the initial physical prior does not fix the graph topology.

Furthermore, a distance matrix among the sensors can be obtained, *d_ij_*. To make the distance matrix *d_ij_* linearly separable, the Gaussian kernel function is used to threshold the distance matrix *d_ij_*, and an initial adjacency matrix of the graph structure is generated *K_ij_*:

(4) $$K_{\mathrm{ij}}=\left\{\begin{array}{l} 1,i=j\\ \exp\left(-\frac{d_{\mathrm{ij}}^{2}}{2\delta^{2}}\right),i\neq j\;\mathrm{and}\;d_{\mathrm{ij}}\geq \tau \\ 0,\mathrm{otherwise} \end{array}\right.$$

where, *δ* is the degree of dispersion of the Gaussian distribution, *τ* is the threshold, which is used to control the distribution density of the adjacency matrix.

### 2.2. Time and Space Feature Extraction

The Graph convolutional network is adopted to perform graph computation on the physical prior adjacency matrix *K*, so as to extract spatial interaction information among individual sensor nodes and generate the feature map 𝒢 embedded with spatial information. The formula for the graph convolutional layer is given as:

(5) $$𝒢=\mathrm{GCN}\left(X,K\right)=\sigma \left({\hat{D}}^{-\frac{1}{2}}\tilde{K}{\hat{D}}^{-\frac{1}{2}}XW_{g}\right)$$

where *X* is the input original features, GCN is the Graph Convolutional Network, $\tilde{K}=K+I_{N}$, *I_N_* is the identity matrix, $\tilde{K}$ is the adjacency matrix with self-loops introduced, $\hat{D}$ is the degree matrix, *W_g_* is the learnable weight matrix of the GCN, *σ* is the nonlinear activation function.

To enhance critical features, the model introduces channel attention and spatial attention. Channel attention is utilized to implement global pooling on the feature map 𝒢 ∈ *ℝ^C^*^×^*^H^*^×^*^W^* output from the GCN. A channel weight vector *w_c_* is generated via fully connected layers and activation functions, which adaptively learns sensor channels that are more significant for the current degradation state and suppresses interference from noisy channels. An updated feature map 𝒢′ ∈ *ℝ^C^*^×^*^H^*^×^*^W^* is thereby obtained:

(6) $$\begin{array}{l} w_{c}=\mathrm{Sigmoid}\left(\mathrm{MLP}\left(\mathrm{AvgPool}\left(𝒢\right)+\mathrm{MaxPool}\left(𝒢\right)\right)\right)\\ {𝒢}^{\prime }=𝒢⊗w_{c} \end{array}$$

where MLP is the Multi-Layer Perceptron, *AvgPool*(·) is the Global average pooling layer, *MaxPool*(·) is the Global Max Pooling Layer, ⊗ is the element-wise multiplication.

Subsequently, the weights of the feature space positions are calculated using the spatial attention graph. The information of the key sensor nodes is extracted in the spatial dimension to obtain the feature map 𝒢″ ∈ *ℝ^C^*^×*H*×*W*^ with enhanced spatial expression ability. The operation process is as follows:

(7) $$\begin{array}{l} w_{s}=\mathrm{Sigmoid}\left(f^{k\times k}\left(\mathrm{AvgPool}\left({𝒢}^{\prime }\right);\mathrm{MaxPool}\left({𝒢}^{\prime }\right)\right)\right)\\ {𝒢}^{\prime \prime }={𝒢}^{\prime }⊗w_{s} \end{array}$$

where, *f^k^*^×*k*^ is the convolution calculation with a convolution kernel of size *k*.

For complex multi-component systems, damage effects become more pronounced as the operating state evolves over time. To strengthen the temporal representation capability of feature extraction, the spatial graph features enhanced by dual-attention mechanisms are processed via temporal convolution. A temporal convolutional network (TCN) is employed to capture the long-term temporal dependencies of the features. The TCN consists of multiple residual blocks. The attention-weighted features 𝒢″ are fed into stacked multi-layer residual temporal convolution blocks (ResBlock). Each residual block is stacked with two dilated causal convolutional layers, and the input-output relationship can be expressed as:

(8) $$\hat{X}={\mathrm{ResBlock}}_{n}\left(\cdots {\mathrm{ResBlock}}_{1}\left({𝒢}^{\prime \prime }\right)\right)$$

(9) $$\begin{array}{l} {\mathrm{ResBlock}}_{i}\left({𝒢}^{\prime \prime }\right)=\mathrm{ReLU}\left(\mathrm{ReLU}\left(W_{2}\cdot z_{t_{i}}\right)+W_{s}\cdot {𝒢}_{t_{i}}^{\prime \prime }\right)\\ z_{t_{i}}=\mathrm{CausalConv}\left(\mathrm{ReLU}\left(W_{1}\cdot z_{t_{i}}^{\prime }\right)\right)\\ z_{t_{i}}^{\prime }=\mathrm{CausalConv}\left({𝒢}_{t_{i}}^{\prime \prime },{𝒢}_{t_{i-1}}^{\prime \prime },{𝒢}_{t_{i-2}}^{\prime \prime },{𝒢}_{t_{i-3}}^{\prime \prime },\cdot \cdot \cdot \right) \end{array}$$

where, ResBlock*_i_*(⋅) is the residual block, ReLU(⋅) is the activation function, *CausalConv*(⋅) is the causal convolution block, *W*_1_,*W*_2_,*W_s_* is the weight coefficient, ${𝒢}_{t_{i}}^{\prime \prime }$ is the characteristic information at time point *t_i_*.

### 2.3. Variational Graph Structure Information Bottleneck and Prediction

Masking is performed on the initial graph features $\hat{X}$ processed by the spatiotemporal feature extraction module to eliminate irrelevant noise. The probabilistic adjacency matrix is relearned based on the masked features for robustness enhancement, that is:

(10) $$\begin{array}{l} \tilde{X}=\hat{X}⊙M_{\mathrm{feat}}\\ \tilde{K}=\tilde{X}⊙M_{\mathrm{graph}} \end{array}$$

where, ⊙ is the element-wise multiplication, *M_feat_* is the feature mask processing, *M_graph_* is the probabilistic graph structure processing.

The information bottleneck subgraph is constructed based on the $\tilde{X}$ and the new probability adjacency matrix $\tilde{K}$, that is:

(11) $${𝒢}_{m}=\mathrm{GCN}\left(\tilde{X},\tilde{K}\right)$$

The graph structure is mapped to the parameters of the latent variable distribution using the GIN encoder and global pooling. It is assumed that the latent variables follow a Gaussian distribution:

*μ*(𝒢*_m_*),*σ*(𝒢*_m_*) = Encoder(𝒢*_m_*) (12)

*P*(*Z_IB_*|𝒢*_m_*) = 𝒩(*μ*(𝒢*_m_*),diag(*σ*^2^(𝒢*_m_*))) (13)

In order to enable the model to perform backpropagation, the reparameterization technique is employed to sample noise from the standard normal distribution *ε*∼𝒩(0,*I*), thereby obtaining the latent variables *Z_in_*:

*Z_in_* = *μ*(𝒢*_m_*) + *σ*(𝒢*_m_*)⊙*ε* (14)

where, *ε* is the randomness that enables the model to represent the uncertainty of the data.

Calculate the global self-attention for the latent variable *Z_in_* to further capture the long-distance dependencies among the variables:

(15) $$\mathrm{Attention}\left(Q,K,V\right)=\mathrm{Softmax}\left(\frac{QK^{T}}{\sqrt{d_{k}}}\right)V$$

where, *Q* = *Z_in_W_Q_*, *K* = *Z_in_W_K_*, *V* = *Z_in_W_V_*, *d_k_* is the scaling factor.

The self-attention output result *Z_attn_* is compressed in terms of dimensions through a fully connected network, and finally the predicted RUL $\overset{⏜}{y}$ is obtained:

(16) $${\hat{y}}_{\mathrm{RUL}}={\mathrm{MLP}}_{\mathrm{FC}}\left(Z_{\mathrm{attn}}\right)$$

The training objective of the model follows the principle of the information bottleneck, which is to maximize the mutual information regarding RUL while minimizing the mutual information regarding the input graph structure:

minℒ*_IB_* = −*I*(𝒢*_m_*;*Y*) + *β*_1_ ⋅ *I*(𝒢*_m_*;𝒢) (17)

where *I*(𝒢*_m_*;*Y*) is the mutual information between the latent graph and the RUL label, *I*(𝒢*_m_*;𝒢) is the mutual information between the latent map and the original spatial feature map, *β*_1_ is the information compression balance hyperparameter.

In order to simultaneously fit the RUL prediction values and the distribution of the constrained latent space, the overall loss function of the model ℒ*_total_* consists of two parts: the RUL prediction loss ℒ*_MSE_* and KL divergence loss ℒ*_KL_*. The RUL prediction loss is usually measured by the mean squared error to quantify the difference between the true value *y* and the predicted value $\hat{y}$, that is:

(18) $$L_{\mathrm{MSE}}=\frac{1}{M}{\sum_{i=1}^{M}\left\|y_{i}-{\hat{y}}_{i}\right\|}^{2}$$

The KL divergence loss is to constrain the posterior distribution *q_ϕ_*(*Z_in_*|𝒢*_m_*) output by the encoder to be as close as possible to the standard normal prior distribution *p*(*Z*)∼𝒩(0,*I*):

(19) $$L_{\mathrm{KL}}=\mathrm{KL}\left[q_{\phi }\left(Z_{\mathrm{in}}\left|{𝒢}_{m}\right.\right)\left\|𝒩\left(0,I\right)\right.\right]=-\frac{1}{2}\sum_{j=1}^{J}\left(1+\log\left(\sigma_{j}^{2}\right)-\mu_{j}^{2}-\sigma_{j}^{2}\right)$$

The final total loss function is:

ℒ*_total_* = ℒ*_MSE_* + *β*_2_ ⋅ ℒ*_KL_* (20)

where, *β*_2_ is the weight coefficient for balancing the losses of the two parts, *μ_j_* and *σ_j_* are the mean and variance of the Gaussian distribution, respectively.

## 3. Data Verification

In the model validation phase, this paper adopts the Commercial Modular Aero-Propulsion System Simulation (C-MAPSS) dataset provided by NASA, Washington D.C., USA [32]. This dataset contains four subsets: FD001, FD002, FD003, and FD004, which simulate different operating conditions. Among them, FD001 and FD003 correspond to a single operating condition with simulations of 100 engines, whereas FD002 and FD004 cover six operating conditions with simulations of 260 engines.

**Figure 3:**
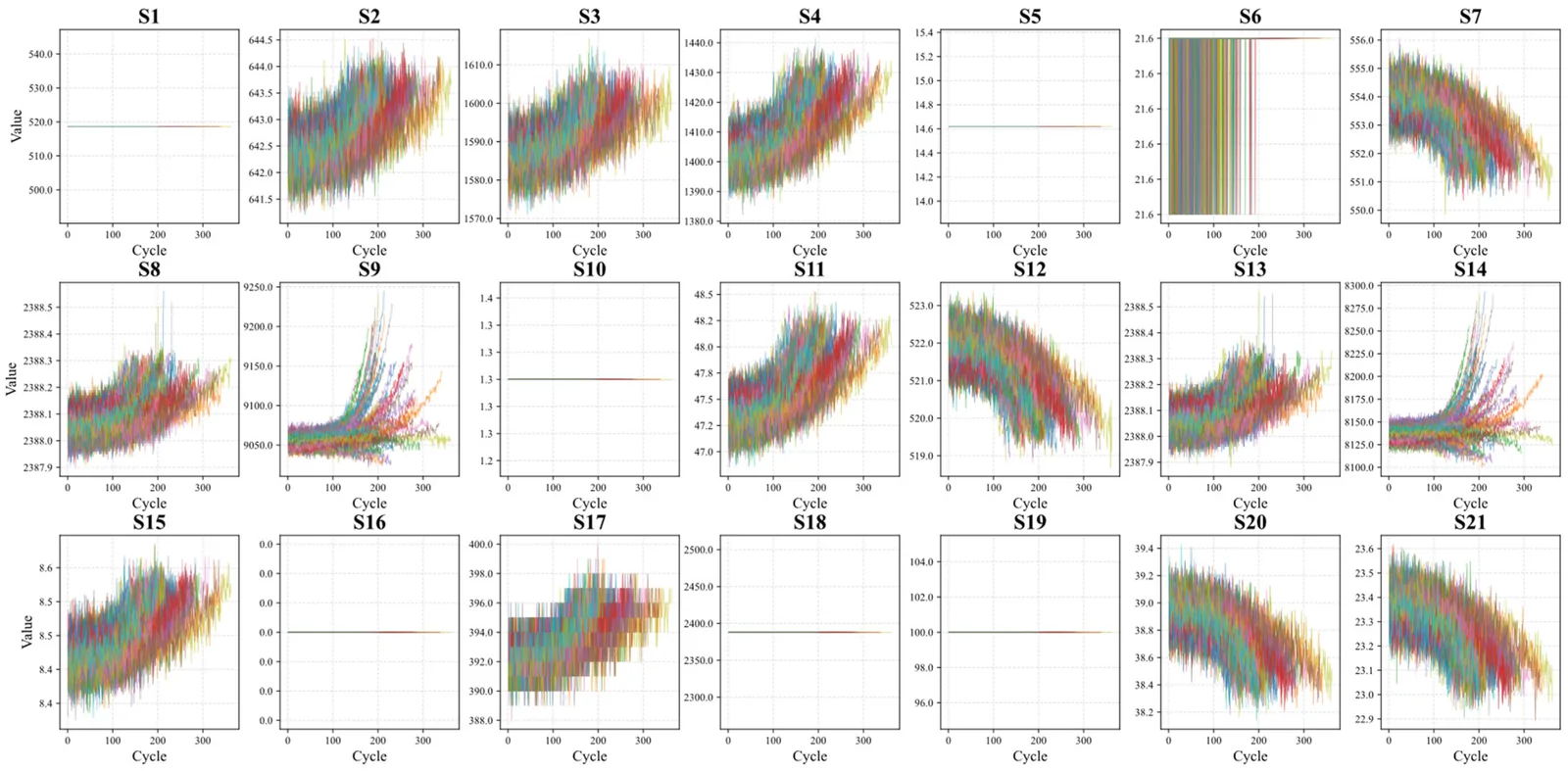
Sensor Monitoring Data.

Four aero-engine subsets are employed for experimental simulation studies of the proposed model. The sensor measurements collected during the operation of aero-engines in the C-MAPSS dataset exhibit diverse variations, reflecting the degradation trends of different engine performance parameters. To improve the prediction accuracy of aero-engine remaining useful life, sensor channels that maintain constant values throughout engine degradation (s1, s5, s6, s10, s16, s18, s19) are removed during data preprocessing. The 14-dimensional sensor measurements showing evident degradation trends are adopted as the raw features for RUL prediction, namely s2, s3, s4, s7, s8, s9, s11, s12, s13, s14, s15, s17, s20, and s21, as illustrated in Figure 3. The abscissa “Cycle” in Figure 3 denotes the flight mission cycle count defined by the C-MAPSS dataset, rather than the rotor rotation period. One Cycle corresponds to one complete simulated flight mission covering the full procedure of take-off, climb, cruise, and landing. Upon completion of each Cycle, service damage accumulates on engine components. As the Cycle number increases continuously, component performance degrades progressively until the failure threshold is reached and the simulation terminates. Individual engines exhibit obvious differences in the total number of mission cycles corresponding to their full service lives. Within the NASA C-MAPSS simulation framework, simulation termination is triggered by the system-level performance degradation threshold, instead of setting separate failure limits for each individual component. Each engine repeatedly executes flight mission cycles, and the health degradation coefficients of components including fans, compressors, and turbines are updated after every cycle. When system-level performance metrics such as overall thrust and fuel consumption rate decay to the permissible lower limit, the engine is deemed to reach its end of life and the simulation stops. The performance degradation of different engines can be dominated by distinct component failures, which enables the simulation of heterogeneous degradation processes among multiple components. The public dataset does not export component-specific internal failure thresholds or failure mode labels generated during simulation; only sensor time-series data and the total failure cycle count for each engine are released. Accordingly, the ground truth label of sample remaining useful life is calculated at the system level: RUL_true_ = Total failure cycles of engine Current flight Cycle index. Following common practice in this research community, a RUL truncation threshold of 125 is adopted. The RUL value is capped at 125 for engines in their early service stage with remaining life exceeding 125 cycles, so as to focus on prediction performance in the late degradation phase. Hyperparameter sensitivity tests also verify the rationality of this life threshold setting. Restricted by the publicly available dataset, this study performs system-level remaining useful life prediction and does not distinguish component failure types corresponding to different engines. Further research on RUL prediction under diverse failure modes can be conducted if datasets annotated with component-level failure information become available in the future. To boost model training efficiency and convergence speed, the min-max normalization approach is utilized to preprocess the sensor data. The specific calculation formulation is given as follows:

(21) $$x_{\mathrm{norm}}=\frac{x-\min\left(x\right)}{\max\left(x\right)-\min\left(x\right)}$$

where, *x* is the original data, min(*x*) is the minimum data value, max(*x*) is the maximum data value, *x_norm_* is the normalized data.

The paper uses two commonly used evaluation indicators for predicting the RUL of aero-engines: Root Mean Square Error (RMSE) and scoring functions. The RMSE measures the average deviation between the predicted and actual values. The smaller the RMSE, the higher the prediction accuracy. The specific calculation formula is as follows:

(22) $$\mathrm{RMSE}=\sqrt{\frac{1}{N}\sum_{i=1}^{N}{\left(\mathrm{RU}L_{i}-\mathrm{Tru}e_{i}\right)}^{2}}$$

where *RUL_i_* is the RUL prediction value, *True_i_* is the RUL true value, *N* is the sample size. According to the degradation law of an aero-engine, the RUL of an aero-engine remains basically unchanged at the initial stage of operation. Combined with the research conclusions of other scholars, the maximum number of cycles of aero-engine life is set to 125 [33].

According to the characteristics of aero-engine degradation, early prediction is usually better than late prediction. However, it is difficult to distinguish the importance of early prediction error and late prediction error in the RMSE evaluation index. The scoring function can be designed so that the penalty value increases exponentially with error magnitude. The specific calculation formula is as follows:

(23) $$\mathrm{Score}=\left\{\begin{array}{c} \sum_{i=1}^{n}e^{-\left(\frac{d}{a_{1}}\right)}-1,d<0\\ \sum_{i=1}^{n}e^{\left(\frac{d}{a_{2}}\right)}-1,d\geq 0 \end{array}\right.$$

where, *d* = *RUL_i_* − *True_i_*, *a*_1_ = 10, *a*_2_ = 13.

Besides the point prediction metrics RMSE and Score, Prediction Interval Coverage Probability (PICP) and Prediction Interval Normalized Average Width (PINAW) are introduced to evaluate the probabilistic prediction results and assess the model’s uncertainty quantification capability. PICP represents the proportion of samples whose ground truth RUL values fall within the 95% prediction confidence intervals output by the model among all test samples, and the ideal PICP should be close to the 95% confidence level. PINAW measures the average width of prediction intervals. Under the premise of guaranteeing coverage rate, a smaller PINAW indicates a tighter interval estimation.

(24) $$\mathrm{PICP}=\frac{1}{N}\sum_{i=1}^{N}I[{\mathrm{RUL}}_{\mathrm{low},i}\leq {\mathrm{RUL}}_{\mathrm{true},i}\leq {\mathrm{RUL}}_{\mathrm{up},i}]$$

(25) $$\mathrm{PINAW}=\frac{1}{N\cdot ({\mathrm{RUL}}_{\max}-{\mathrm{RUL}}_{\min})}\sum_{i=1}^{N}\left({\mathrm{RUL}}_{\mathrm{up},i}-{\mathrm{RUL}}_{\mathrm{low},i}\right)$$

where N is the total number of samples in the test set. *I*[⋅] is the indicator function, taking 1 if the condition is met and 0 otherwise. RUL_low__,*i*_ and RUL_up__,*i*_ are the upper and lower limits of the 95% confidence interval of the i sample. RUL_max_ and RUL_min_ are the maximum and minimum values of the RUL label. This paper constructs the 95% confidence interval based on sampling from the latent variable Gaussian distribution *z*∼𝒩(*μ*,*σ*^2^) output by the variational module.

To validate the effectiveness of the proposed model, all simulation experiments are conducted on laptops with Python3.9, torch2.8.0, CUDA13.0, Intel Core Ultra 9 275HX CPU, and NVIDIA GeForce RTX 5070 GPU.

## 4. Result Analysis

During model validation, sensitivity analysis is first performed on the hyperparameter settings of the network prediction model. The investigated hyperparameters include different batch sizes, TCN layer numbers, time scale lengths, life threshold values, and loss weight coefficients. The influences of these hyperparameters on aero-engine RUL prediction are analyzed to determine the optimal hyperparameter configuration for the prediction model. Secondly, the RUL prediction experiments for aero-engines are carried out under the optimal hyperparameter configuration, and the prediction results are compared and analyzed with those obtained by existing methods. Finally, ablation experiments are designed to analyze the contribution of each functional module of the proposed model to the improvement of aero-engine RUL prediction accuracy.

### 4.1. Model Parameter Sensitivity Analysis

**Figure 4:**
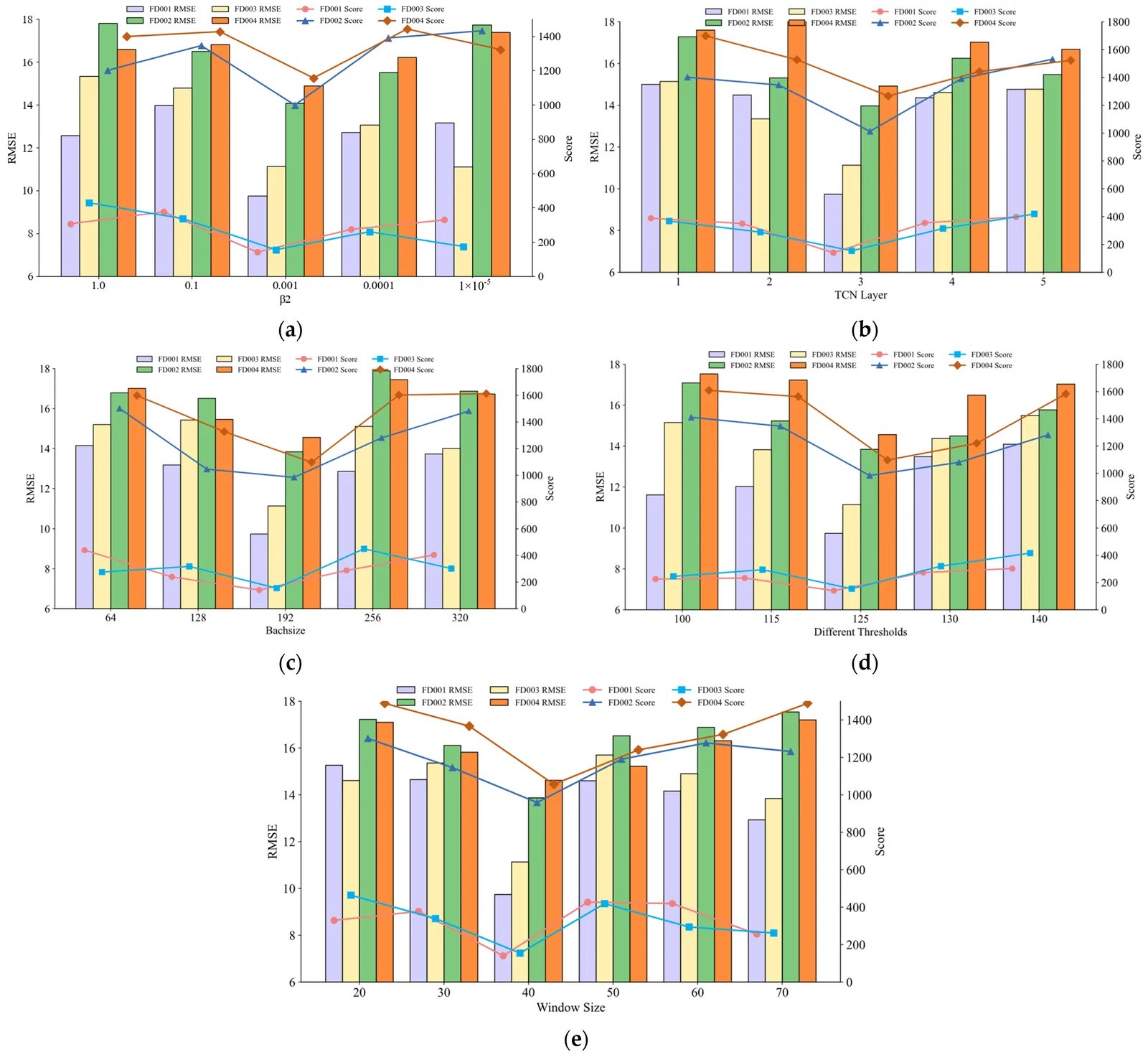
Model hyperparameter settings: (**a**) Different *β*_2_; (**b**) Different TCN layers; (**c**) Different batch sizes; (**d**) Different truncation thresholds; (**e**) Different time windows.

To obtain the optimal hyperparameter configuration of the prediction model, simulation experiments and result analyses are carried out for hyperparameters including different batch sizes, TCN layer numbers, time scale lengths, life threshold values, and loss weight coefficients. To verify the universality of the hyperparameters under single-condition and multi-condition scenarios, simulation analysis is conducted simultaneously using the single-condition datasets FD001 and FD003, as well as the multi-condition datasets FD002 and FD004. The distributions of RMSE and error score values for aero-engine RUL prediction under diverse hyperparameter settings are obtained, as shown in Figure 4a–e.

Figure 4 illustrates the error distributions of aero-engine RUL predictions on the FD001 and FD003 datasets under different hyperparameter configurations of the prediction model. Among these subplots, Figure 4a shows the prediction error distributions when the weight coefficient of the model loss function takes different magnitudes. The results reveal that the prediction errors and score values of both datasets reach their minimum when the loss function weight balance coefficient is on the order of *β*_2_. By contrast, larger or smaller values than *β*_2_ lead to increased prediction errors on both datasets. This indicates that neither excessively large nor excessively small values are favorable for loss computation and iterative convergence of the prediction model. Figure 4b presents the prediction error distributions when the number of residual layers within the temporal convolution module ranges from 1 to 5. The proposed model achieves optimal prediction accuracy on both datasets with three residual layers. Prediction errors increase when the residual layer number is set to 1 or 5. This phenomenon suggests that insufficient temporal feature extraction occurs with too few TCN layers, whereas redundant feature extraction arises with excessive TCN layers, resulting in enlarged prediction errors. Figure 4c depicts the prediction error distributions under batch size settings of 64, 128, 192, 256, and 320. The results demonstrate that the RMSE and error score values peak at batch sizes of 64 and 320, implying that both overly small and overly large batch size configurations degrade prediction accuracy. The minimal RMSE and score values are obtained at a batch size of 192. Figure 4d shows the prediction error distributions under life truncation threshold values of 100, 115, 125, 130, and 140. The results indicate that the model yields the lowest RMSE and score value at a truncation threshold of 125, which is consistent with the widely adopted threshold of 125 reported in most existing literature. Figure 4e gives the prediction error distributions with sliding time window sizes of 20, 30, 40, 50, 60, and 70. Larger RMSE and error score values are observed for window sizes of 20 and 30, demonstrating that small sliding time windows impair the model’s capability to capture temporal dependency information from sensor measurements. The prediction errors for both datasets attain the minimum at a window size of 40. Further enlargement of the sliding time window causes the prediction error to rise again. It can therefore be concluded that model prediction accuracy does not continuously improve with the increase in sliding time window size. It can be observed from the sensitivity results on the multi-condition datasets FD002 and FD004 that although the absolute values of overall RMSE and Score metrics for FD002 and FD004 are considerably higher than those for FD001 and FD003, the optimal hyperparameter combination (loss weight coefficient *β*_2_ is 10^−3^, Batch size is 192, TCN layer number is 3, sliding time window is 40, RUL truncation threshold is 125) also yields the best performance on multi-condition datasets, which is consistent with the optimal parameters obtained from single condition datasets. When hyperparameters deviate from this optimal configuration, increased prediction errors can likewise be observed for FD002 and FD004. This result demonstrates that the hyperparameter combination determined in this work is not only suitable for single-condition scenarios but also effective for complex multi-condition cases. The fundamentally higher prediction metrics of FD002 and FD004 stem from the six distinct operating conditions contained in these datasets, which introduce notable operating condition distribution shift. This is an inherent characteristic of the datasets rather than being caused by inappropriate hyperparameter settings. Accordingly, this set of optimal hyperparameters is adopted uniformly for all subsequent experiments in this paper.

### 4.2. Comparison of Model Prediction Results with Existing Methods

**Figure 5:**
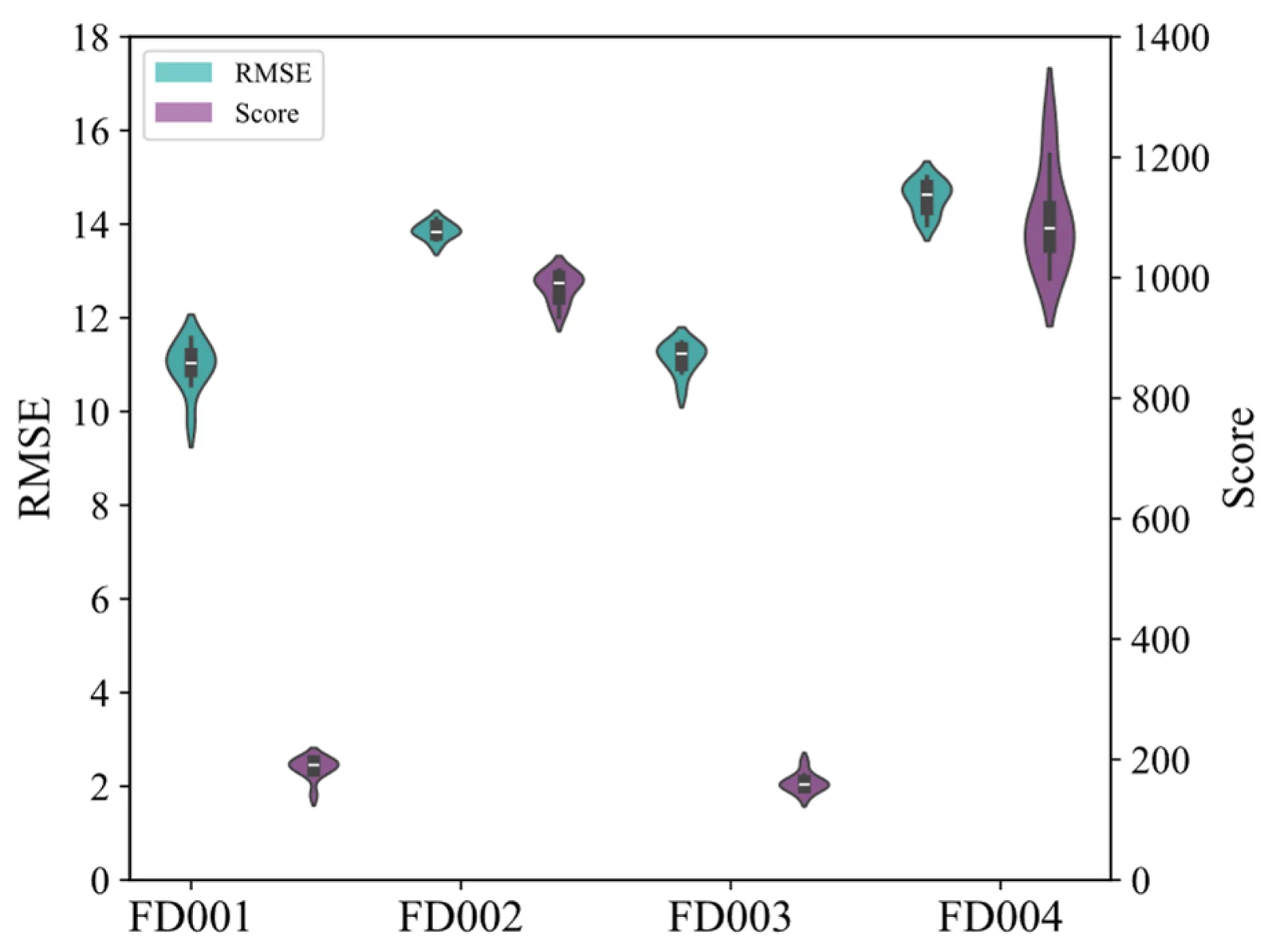
Violin plot showing the distribution of prediction error for Model 10 times.

To verify the superiority of the proposed prediction model, the prediction results of the presented method are compared with those of existing methods using aero-engine datasets under identical operating conditions. To guarantee the validity of result comparison, the data preprocessing strategy, life truncation threshold, and error evaluation function adopted in this work are consistent with those used in the compared references. To eliminate the bias induced by random weight initialization or random dropout parameters within machine learning network architectures, 10 independent repeated simulation experiments are conducted on the four datasets using the proposed model, and the average values are recorded. The corresponding statistical results are listed in Table 1, and Figure 5 illustrates the error distribution of the ten prediction runs. Table 2 summarizes the RMSE and error score values obtained by the proposed method and the comparative approaches from the literature.

Table 1 summarizes the prediction results of 10 independent repeated simulation experiments performed by the model on the four datasets FD001–FD004, and Figure 5 presents the corresponding violin plot error distributions. As can be observed from the quantitative results in Table 1, the average RMSE for RUL prediction of the proposed model over the four datasets are 10.96, 13.84, 11.14, and 14.56, respectively, and the corresponding average error score values are 185.83, 983.76, 161.02, and 1097.73. As indicated by the violin plot distributions in Figure 5, the violin shapes of RMSE and error score values obtained from ten prediction runs on all four datasets are flattened, demonstrating that the prediction results are densely distributed. For the FD001 dataset, the minimum and maximum RMSE are 9.74 and 11.57, with a range of 1.83. For the FD002 dataset, the minimum and maximum RMSE are 13.51 and 14.12, yielding a range of 0.61. For the FD003 dataset, the minimum and maximum error score values are 136.6 and 196.2, with a range of 59.6. For the FD004 dataset, the minimum and maximum error score values are 998.71 and 1268.45, with a range of 269.74. In conclusion, the distributions of RMSE and error score values for FD001-FD004 all fall within an acceptable range, which verifies the favorable prediction stability of the proposed model.

**Figure 6:**
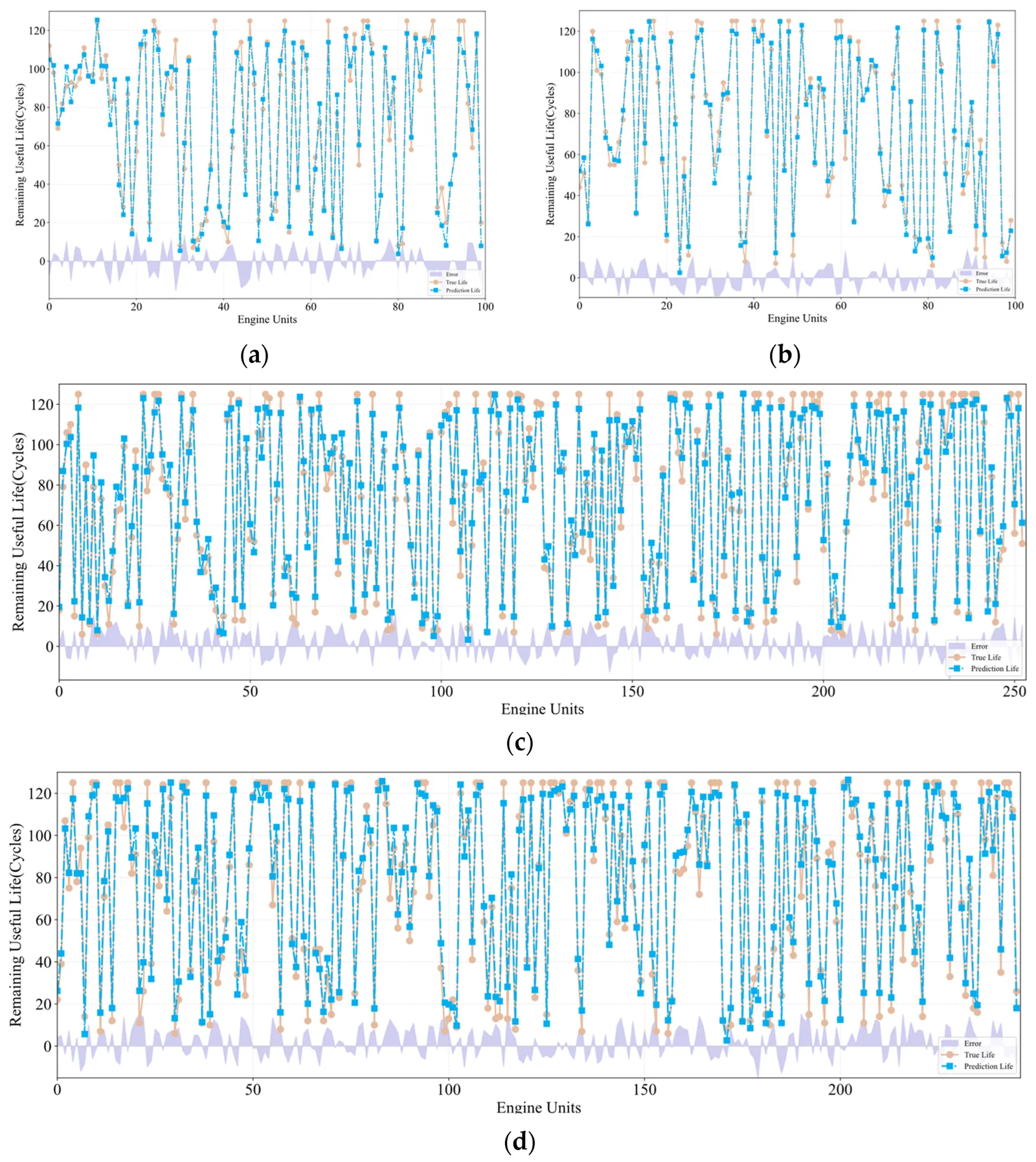
The RUL prediction results of the model in this paper on four datasets: (**a**) FD001 Dataset; (**b**) FD003 Dataset; (**c**) FD002 Dataset; (**d**) FD004 Dataset.

Table 2 compares the RUL prediction results between the proposed model and existing methods across the four datasets. The results show that, except for slightly larger RMSE on FD001 and FD003 compared with KEST-GCN (2025), the proposed model achieves superior RMSE and error score values on the remaining datasets relative to the comparative approaches from the literature. Compared with KEST-GCN (2025), the proposed model introduces the variational information-bottleneck theory on the basis of the physics-embedded graph convolutional network framework, which strengthens the model’s capability to filter relevant information. Although the proposed model yields slightly higher RMSE for single-condition datasets FD001 and FD003, our method obtains better error-score values. Moreover, the proposed model significantly outperforms KEST-GCN (2025) on FD002 and FD004 under complex operating conditions. This demonstrates that the presented model improves the capability of existing approaches for RUL prediction under complex working conditions. When compared with other literature methods, the proposed model obtains better RMSE and error score values over all four datasets. It indicates that the physical prior information incorporated into the graph structure effectively enhances the neural network’s state representation capability for multi-component systems and further improves RUL prediction accuracy. Figure 6a–d illustrate the RUL prediction distributions of the proposed model for the aero-engine datasets FD001-FD004. The *X*-axis denotes the engine index, and the *Y*-axis represents the RUL value. The results reveal that the predicted RUL values of the proposed model agree well with the ground truth values for all four datasets. The absolute RUL prediction error for each engine index is less than 20, which confirms the favorable RUL prediction stability of the proposed model for aero-engines.

### 4.3. Ablation Experiment

**Figure 7:**
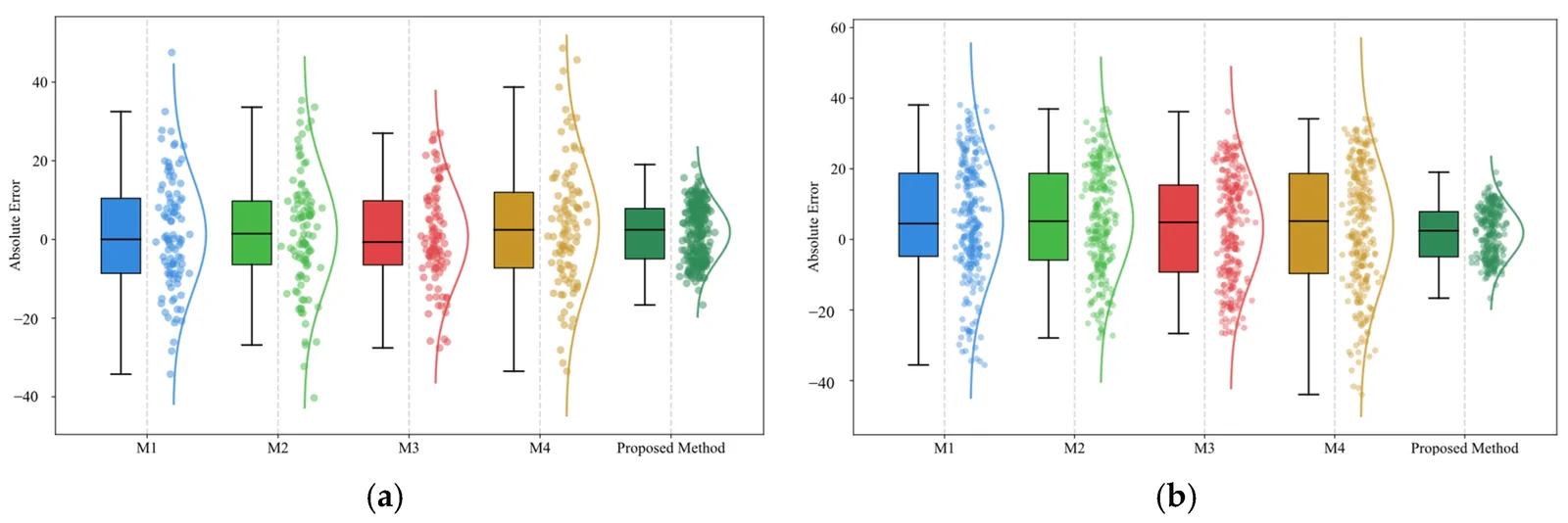
Distribution of absolute error in the ablation comparative experiment: (**a**) FD001 Dataset; (**b**) FD002 Dataset.

To verify the effectiveness and contribution of functional modules, including physical prior information, spatiotemporal feature extraction, and variational information filtering for RUL prediction, ablation simulation experiments are carried out for the proposed method. The configurations of the ablated models are summarized in Table 3. M1 removes the physical prior topological matrix from the complete model framework and adopts a random matrix instead. M2 excludes the dual attention mechanism for spatial feature extraction. M3 discards the temporal convolutional network for temporal feature extraction. M4 eliminates the variational information bottleneck module for relevant information filtering. The full proposed model integrates all the above-mentioned functional modules. For the convenience of experimental implementation, FD001 under a single operating condition and FD002 under a complex operating condition are selected for ablation studies. Table 4 lists the RMSE and error score values obtained from ablation experiments on these two datasets, and Figure 7 presents the distribution of absolute prediction errors.

Table 4 presents the prediction results of the ablation comparison experiments. Compared with the prediction performance of the full proposed model, M1 and M4 yield substantially larger prediction errors and error score values under both single and complex operating condition datasets, and their errors are also higher than those of M2 and M3. This reveals that the physical prior topological matrix and variational information filtering module exert the most significant influences on aero-engine RUL prediction. Introducing the physical prior topological matrix at the front end and the relevant information filtering mechanism at the back end of the network can effectively enhance the physical interpretability and complex condition processing capability of the neural network. The prediction errors of M2 and M3 are both higher than those of the full model, demonstrating that the dual attention spatial feature extraction module and temporal feature extraction module contribute to improving the model’s spatiotemporal feature representation capability. Figure 7 shows the absolute prediction error distributions of all ablation variants. The results indicate that M1, M2, and M4 exhibit a wide spread of scattered absolute error points, while M3 presents relatively concentrated error distributions. By comparison, the scattered error points of the proposed full model are the most compact. This suggests that M1, M2, and M4 suffer from poor prediction stability, whereas the proposed model achieves favorable stability. Accordingly, the physical prior information module and variational information filtering module make major contributions to boosting the RUL prediction accuracy for aero-engines.

### 4.4. Quantitative Analysis of Uncertainty in Prediction

The variational information bottleneck module encodes degradation features into a Gaussian latent variable distribution, where the variance term *σ* quantifies the degree of prediction uncertainty for each sample. To verify the uncertainty-modeling performance of the proposed model, the PICP (Prediction Interval Coverage Probability) and PINAW (Prediction Interval Normalized Average Width) metrics corresponding to the 95% confidence interval are calculated on the FD001-FD004 datasets. The experimental results are presented in Table 5.

As can be seen from the results in Table 5, the PICP values of single-condition datasets FD001 and FD003 reach 92.47% and 91.85%, respectively, which are very close to the theoretical confidence level of 95%. Owing to complex operating conditions and large fluctuations in data distribution, the PICP values of multi-condition datasets FD002 and FD004 decrease slightly but remain around 90%, accompanied by increased PINAW values. This observation conforms to the objective law of elevated prediction uncertainty under multi-condition scenarios. Further inspection at the sample level reveals that in the early service stage of an aero-engine, equipment degradation is mild and sensor signals are stable, leading to a small latent variable variance *σ* and narrow prediction intervals. As cycles accumulate continuously, the engine gradually approaches failure, sensor noise rises, and degradation stochasticity intensifies. Accordingly, the latent variable variance *σ* increases remarkably, and the prediction intervals become wider. This phenomenon is consistent with the actual physical degradation law of aero-engines: in the pre-failure stage, the stochasticity of equipment degradation behavior grows, and prediction uncertainty is expected to increase.

## 5. Conclusions

This paper proposes a RUL prediction method for multi-component systems based on physical priors and a variational spatiotemporal graph convolutional network. An aero-engine is taken as a typical case for simulation studies. Firstly, according to the physical connection architecture of a multi-component aero-engine, non-Euclidean sensor measurements are transformed into graph-structured data embedded with physical topological priors, so as to extract physical dependency information among sensors. Secondly, the dual attention mechanism is adopted to identify spatial positions and sensor channels that contribute most to RUL prediction within graph features. The temporal convolution module is further employed to capture temporal dependency information of features and strengthen the model’s perception capability for spatiotemporal characteristics. Finally, the graph features processed by the spatiotemporal feature extraction module are fed into the variational information bottleneck module. The masking mechanism is utilized to eliminate irrelevant noise, screen out information correlated with life-related features, and remove redundant information irrelevant to life degradation. The information-filtered features are then imported into a multi-layer perceptron network to achieve a scalar representation for aero-engine RUL prediction. Four public aero-engine datasets released by NASA are adopted to validate the proposed model. The prediction results are compared with those of the existing literature methods. The proposed method yields lower RMSE and error score values, which verifies the superiority of the presented approach and provides a new perspective for RUL prediction research of multi-component systems. Nevertheless, the proposed model still has limitations regarding the generality and dynamics of physical prior construction. Future work will focus on developing generalized physical prior models applicable to diverse systems.

## Figures and Tables

**Table 1:** 10 Prediction Results of FD001-FD004 Data.

Calculation Number	FD001		FD002		FD003		FD004	
	RMSE	Score	RMSE	Score	RMSE	Score	RMSE	Score
1	11.23	198.7	13.51	958.6	11.33	162.4	14.62	1074.51
2	10.91	193.0	13.78	934.85	11.48	196.2	14.53	1103.78
3	9.74	140.3	13.67	989.76	11.13	154.5	14.26	1056.78
4	11.01	196.7	14.02	1004.5	10.95	153.9	14.92	1204.31
5	10.85	188.8	13.97	986.54	10.40	136.6	13.98	998.71
6	11.20	181.5	13.82	993.64	11.20	174.2	14.10	1009.78
7	11.06	202.0	14.12	1012.34	11.46	164.4	15.01	1268.45
8	11.55	183.0	13.85	992.81	10.83	150.8	14.78	1121.56
9	10.56	178.2	13.79	958.73	11.27	160.2	14.82	1089.72
10	11.57	196.1	13.92	1005.84	11.35	157.0	14.64	1049.78
Average value	10.96	185.83	13.84	983.76	11.14	161.02	14.56	1097.73

**Table 2:** RMSE and score of the proposed method and other methods.

Methods	FD001		FD002		FD003		FD004	
	RMSE	Score	RMSE	Score	RMSE	Score	RMSE	Score
BDNN (2020) [34]	12.19	267.2	18.49	2007.8	12.07	409.4	19.41	2415.7
AGCNN (2020) [35]	12.42	225.5	19.43	1492	13.39	227.1	21.50	3392
STAF (2022) [36]	11.35	194.4	19.17	2493.1	11.64	224.5	17.73	2760.1
GAT-DAT (2023) [37]	13.83	318.6	14.81	1163.8	14.85	438.5	16.8	1928.6
SCTA (2023) [38]	12.10	207	16.9	1267	12.14	248	21.93	3310
TATFA (2024) [39]	12.21	261.5	15.07	1359.7	11.23	210.2	18.81	2506.3
SACN (2024) [40]	12.8	291.59	19.87	3184.75	14.57	418.89	23.9	7404.11
CATA-TCN (2024) [41]	12.8	234.31	17.61	1361.23	13.16	290.63	21.04	2303.42
ADFG (2025) [42]	11.34	203.12	16.31	1262.06	11.72	211.24	17.15	1327.51
FIT-GSTM (2025) [43]	11.23	183	15.32	1046	11.48	233	16.98	1488
KEST-GCN (2025) [44]	10.77	267.8	16.99	1100.6	9.69	238.7	17.73	2747.1
Proposed Method	10.96	185.83	13.84	983.76	11.14	161.02	14.56	1097.73

**Table 3:** Comparison of Ablation Models.

Model	Physical Priori Topological Matrix	Dual Attention Module	TCN Time Feature Extraction	Variational Information Bottleneck
M1	No	yes	yes	yes
M2	Yes	no	yes	yes
M3	yes	yes	no	yes
M4	yes	yes	yes	no
Proposed method	yes	yes	yes	yes

**Table 4:** Prediction Results of Ablation Comparative Experiment.

Model	RMSE		Score	
	FD001	FD002	FD001	FD002
M1	15.58	18.72	439.3	1898.4
M2	14.74	16.78	357.2	1324.7
M3	13.07	15.84	349.1	1240.5
M4	15.47	18.32	446.5	1846.3
Proposed method	10.96	13.84	185.83	983.76

**Table 5:** The 95% confidence interval results of PICP and PINAW for each dataset.

Datasets	PICP (%)	PINAW
FD001	92.47	0.216
FD002	90.12	0.283
FD003	91.85	0.227
FD004	89.64	0.301

## Data Availability

No new data were created or analyzed in this study. Data sharing is not applicable to this article.

## Abbreviations

The following abbreviations are used in this manuscript:

RUL	Remaining useful life
GNNs	Graph neural networks
GIN	Graph Isomorphism network
GCN	Graph convolutional networks
PHM	Prognostics and Health Management
